# *Listeria monocytogenes* Wall Teichoic Acid Glycosylation Promotes Surface Anchoring of Virulence Factors, Resistance to Antimicrobial Peptides, and Decreased Susceptibility to Antibiotics

**DOI:** 10.3390/pathogens9040290

**Published:** 2020-04-16

**Authors:** Diana Meireles, Rita Pombinho, Filipe Carvalho, Sandra Sousa, Didier Cabanes

**Affiliations:** 1 i3S–Instituto de Investigação e Inovação em Saúde, Universidade do Porto, 4200-135 Porto, Portugal; diana.meireles@ibmc.up.pt (D.M.); rita_pombinho@i3s.up.pt (R.P.); ffchcarvalho@gmail.com (F.C.); srsousa@ibmc.up.pt (S.S.); 2 Group of Molecular Microbiology, IBMC–Instituto de Biologia Celular e Molecular, 4200-135 Porto, Portugal; 3 Cell Biology of Bacterial Infections Group, IBMC–Instituto de Biologia Molecular e Celular, 4200-135 Porto, Portugal

**Keywords:** wall teichoic acid glycosylation, glycosyltransferase, Gram-positive pathogens, antimicrobial peptides, antibiotics

## Abstract

The cell wall of *Listeria monocytogenes* (*Lm*)*,* a major intracellular foodborne bacterial pathogen, comprises a thick peptidoglycan layer that serves as a scaffold for glycopolymers such as wall teichoic acids (WTAs). WTAs contain non-essential sugar substituents whose absence prevents bacteriophage binding and impacts antigenicity, sensitivity to antimicrobials, and virulence. Here, we demonstrated, for the first time, the triple function of *Lm* WTA glycosylations in the following: (1) supporting the correct anchoring of major *Lm* virulence factors at the bacterial surface, namely Ami and InlB; (2) promoting *Lm* resistance to antimicrobial peptides (AMPs); and (3) decreasing *Lm* sensitivity to some antibiotics. We showed that while the decoration of WTAs by rhamnose in *Lm* serovar 1/2a and by galactose in serovar 4b are important for the surface anchoring of Ami and InlB, N-acetylglucosamine in serovar 1/2a and glucose in serovar 4b are dispensable for the surface association of InlB or InlB/Ami. We found that the absence of a single glycosylation only had a slight impact on the sensibility of *Lm* to AMPs and antibiotics, however the concomitant deficiency of both glycosylations (rhamnose and *N*-acetylglucosamine in serovar 1/2a, and galactose and glucose in serovar 4b) significantly impaired the *Lm* capacity to overcome the action of antimicrobials. We propose WTA glycosylation as a broad mechanism used by *Lm,* not only to properly anchor surface virulence factors, but also to resist AMPs and antibiotics. WTA glycosyltransferases thus emerge as promising drug targets to attenuate the virulence of bacterial pathogens, while increasing their susceptibility to host immune defenses and potentiating the action of antibiotics.

## 1. Introduction

*Listeria monocytogenes* (*Lm*) is a major intracellular foodborne bacterial pathogen that causes listeriosis, a human systemic infection [1]. Among zoonotic diseases under European Union (EU)-surveillance, listeriosis is the most severe [2]. *Lm* has the capacity to colonize various niches, from inert and organic matrices to the intestinal lumen where it competes with resident microbiota, translocates across the epithelium, multiplies in phagocytic and non-phagocytic cells, and disseminates via the blood [1,3].

In Gram-positive bacteria, the peptidoglycan (PGN) is densely decorated with glycopolymers such as lipoteichoic acids (LTAs), anchored to the head groups of membrane lipids by a diacylglycerol, and wall teichoic acids (WTAs), which are covalently attached to PGN via phosphodiester linkage [4,5]. WTAs are the most abundant PGN-linked polymers in Gram-positive pathogens such as *Lm* and *Staphylococcus aureus*. They play key functional roles in bacterial physiology, including cation binding, osmotic and heat tolerance, regulation of autolytic activity, cell-shape determination, and phage-binding [6]. Furthermore, WTAs have important roles in Gram-positive bacteria pathogenicity, namely by coordinating the mechanisms required for host infection and colonization, and conferring resistance to antimicrobial peptides (AMPs) and decreased susceptibility to antibiotics [6].

In *Lm*, WTAs are composed of repeated ribitol-phosphate (RboP) subunits, whose hydroxyl groups can be substituted by diverse monosaccharides [4,7]. Specific substitutions are characteristic of specific *Lm* serovars (Sv), namely: l-rhamnose (Rha) and *N*-acetylglucosamine (GlcNAc) in Sv1/2 and d-glucose (Glu), and d-galactose (Gal) in Sv4 [8]. Sv-specific phage resistance and virulence attenuation have been attributed to alterations in WTA-glycosylation (WTA-gly) [9,10,11,12,13,14]. Phage predation depends on specific WTA sugar residues and represents a driving force for *Lm* evolution [15]. Importantly, the majority of listeriosis outbreaks have been associated with Sv1/2 and Sv4b [16].

It was previously demonstrated that *Lm* strain EGDe (Sv1/2) WTA decorated with Rha (WTA-Rha) requires both RmlACBD biosynthetic enzymes and the rhamnosyltransferase RmlT, and confers resistance to the action of AMPs [17]. Importantly, we proved that the increased *Lm* susceptibility to AMPs, in the absence of WTA-Rha, is as a result of an increased cell wall permeability, which results in a faster plasma membrane disruption, with lethal consequences for bacteria. WTA-Rha has been clearly shown to be required for *Lm* virulence, but not for growth in an AMP-defective host [17].

The cell wall of Gram-positive bacteria is also the docking site for proteins covalently bound to PGN or noncovalently retained via the interaction with cell wall components [4]. Numerous *Lm* virulence factors are cell wall-associated proteins [3], including InlB and Ami [18,19]. While the cell wall retention of these proteins was previously shown to be partially dependent on LTA and PGN [20,21], WTA-Rha was proven to be essential for their proper surface anchoring [22]. More recently, the loss of WTA-Gal in Sv4b has been shown to not only prevent phage adsorption, but also to lead to a complete loss of surface-associated InlB and virulence attenuation in vivo [23]. Importantly, glycosylated WTAs have been described as being required for decreased susceptibility to antibiotics in *S. aureus* [24].

Our aim here was to analyze if other WTA glycosylations could have an equivalent role on the surface association of virulence factors and resistance to antimicrobial peptides, and to demonstrate the role of WTA glycosylation in *Listeria* sensitivity to antibiotics.

## 2. Results

### 2.1. WTA-glycosylation Promotes Efficient Surface Association of *Lm* Virulence Factors

Previous studies have shown that WTA-Rha in *Lm* Sv1/2a is required for the surface anchoring of virulence factors, namely Ami and InlB [22]. Similarly, the absence of WTA-Gal in Sv4b causes the complete loss of surface-associated InlB [23]. Given that, besides Rha and Gal, *Lm* WTAs can be modified with GlcNAc or Glu moieties, we wondered whether the association of Ami and InlB to the bacterial surface could be broadly dependent on WTA glycosylation events.

**Figure 1:**
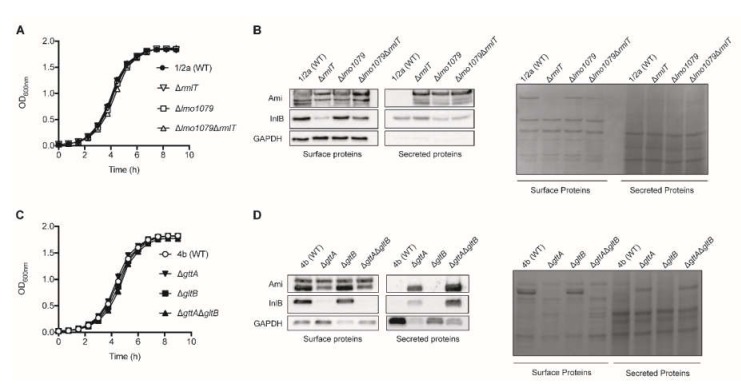
Wall teichoic acid (WTA)-glycosylation promotes surface association of *Listeria monocytogenes* (*Lm*) virulence factors. Growth curves in standard culture conditions in vitro (brain heart infusion (BHI) at 37 °C, with agitation) of the different mutants compared with the isogenic wild type (WT) strains from (**A**) Sv1/2a and (**C**) Sv4b. Data show the optical density values obtained throughout time. Data represent mean ± standard deviation (SD) of three independent experiments. Western blot on extracts of non-covalently cell surface associated and secreted *Lm* proteins obtained from (**B**) Sv1/2a WT, WTA-rhamnosylation deficient (Δ*rmlT*), WTA-acetylglucosylation deficient (Δ*lmo1079*), and deficient for both glycosylations (Δ*lmo1079*Δ*rmlT*) strains, as well as (**D**) Sv4b WT, WTA-galactosylation deficient (Δ*gttA*), WTA-glucosylation deficient (Δ*gltB*), and deficient for both glycosylations (Δ*gttA*Δ*gltB*) strains.*Lm* Glyceraldehyde-3-phosphate dehydrogenase (GAPDH) protein levels, and Ponceau S (Sv1/2a) and Coomassie Brilliant Blue (Sv4b) staining were used as the loading control. Images are representative of at least three independent experiments.

We assessed the surface association and culture media secretion of Ami and InlB in *Lm* Sv1/2a WT, as well as in isogenic mutants deficient for WTA-Rha (Δ*rmlT*), WTA-GlcNAc (Δ*lmo1079*) and for both glycosylations (Δ*lmo1079*Δ*rmlT*) (Figure 1 and Figure S1A). We first confirmed that mutant and wild type (WT) strains grow with comparable rates in a brain heart infusion (BHI) medium at 37 °C, indicating that the absence of any of WTA glycosylation does not impair bacterial growth in pure culture (Figure 1A). The presence of Ami and InlB on the extracts of non-covalently associated surface proteins and on culture supernatants collected from exponential growth cultures was assessed by Western blot analysis. Ami, in its unprocessed (~100 kDa) and processed forms [19], was detected in extracts of surface associated proteins from WT and mutant strains (Figure 1B). As previously described for the WTA-Rha deficient strain (Δ*rmlT*) [22], the levels of surface associated Ami were also slightly reduced in the mutants lacking WTA-GlcNAc (Δ*lmo1079*) or both Rha and GlcNAc (Δ*lmo1079*Δ*rmlT*) as compared to the WT strain (Figure 1B). Strikingly, while Ami was undetectable in the secreted protein extracts from WT Sv1/2a, all of the Ami forms were strongly detected in the culture supernatants from mutants deficient in specific WTA-glycosylations (Figure 1B), demonstrating that both WTA-Rha and WTA-GlcNAc are necessary for full the retention of Ami in a bacterial surface, avoiding its release to the extracellular milieu. As previously reported [22], the WTA-Rha deficient mutant (Δ*rmlT*) show consistently reduced levels of surface-bound InlB and increased InlB amounts in the supernatant fraction compared with WT (Figure 1B). In contrast, the absence of WTA-GlcNAc did not interfere with the InlB surface association or secretion (Figure 1B).

We extended our experimental approach to the *Lm* Sv4b strain, in which WTAs are decorated with Glu and Gal. We used WT Sv4b as well as strains deficient in WTA-Gal (Δ*gttA*), WTA-Glu (Δ*gltB*), and both glycosylations (Δ*gttA*Δ*gltB*). The growth rate of mutant strains was comparable to that of the WT in BHI at 37 °C (Figure 1C). Two forms of Ami were detected in the non-covalently associated surface protein extracts of the WT Sv4b strain, with none of these forms being secreted to the culture medium (Figure 1D). The surface association of the 85 kDa unprocessed Ami remained unchanged in the WTA-Gal (Δ*gttA*) and WTA-Glu (Δ*gltB*) mutants, as well as in the double mutant (Δ*gttA*Δ*gltB*). However, in the strains lacking WTA-Gal (Δ*gttA* and Δ*gttA*Δ*gltB*), the Ami processed form (75 kDa) was decreased in the surface extracts and strongly increased in the secreted extracts (Figure 1D), indicating that WTA glycosylation with Gal is essential for the surface association of the processed form of Ami. As previously reported [23], the absence of WTA-Gal (Δ*gttA* and Δ*gttA*Δ*gltB*) induced an almost complete secretion of InlB (Figure 1D). However, in the absence of WTA-Glu (Δ*gltB*), both Ami and InlB display surface association/secretion patterns similar to the WT strain (Figure 1D).

Altogether, these results indicate that, depending of the serovar, specific WTA-glycosylations are crucial for the efficient surface association of *Lm* virulence factors, namely Ami and InlB.

### 2.2. WTA-glycosylation Promotes *Lm* Resistance to AMPs

**Figure 2:**
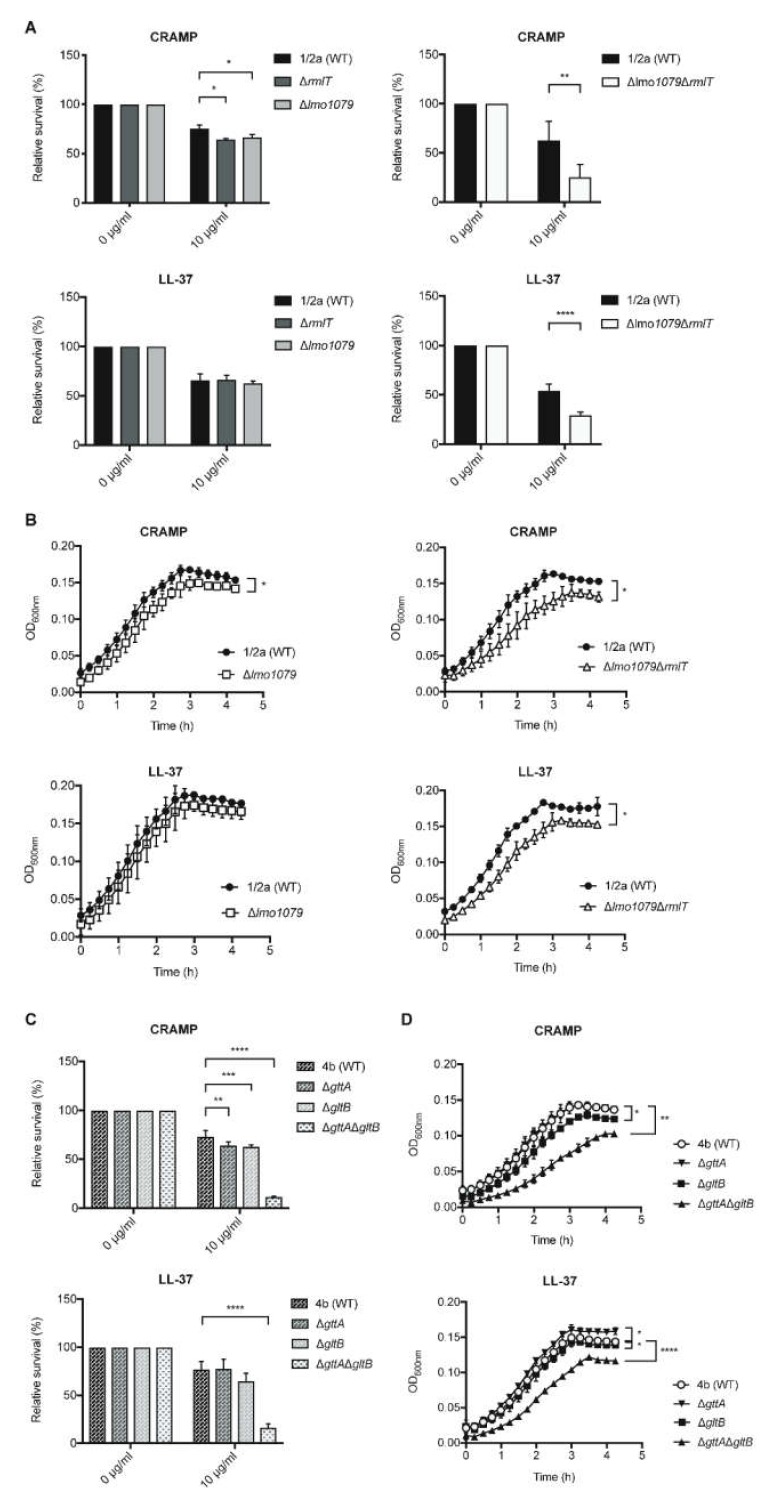
WTA-glycosylation promotes *Lm* resistance against antimicrobial peptides (AMPs). Quantification of viable *Lm* strains, (**A**) Sv1/2a and (**C**) Sv4b, after incubation of the exponential-phase *Lm* strains with the antimicrobial peptides cathelicidin-related antimicrobial peptide (CRAMP) and LL-37 (10 μg/mL). Values from the AMP-treated samples were normalized to untreated controls (set at 100). Data represent mean ± SD of at least three independent experiments. Bacterial growth curves of *Lm* strains (**B**) Sv1/2a and (**D**) Sv4b grown in the presence of CRAMP and LL-37 (10 μg/mL). Data represent mean ± SD of three independent experiments (* *p* < 0.05; ** *p* < 0.01; *** *p* < 0.001; **** *p* < 0.0001).

In *Lm* Sv1/2a, WTA-Rha contributes to resistance against host AMPs [17]. Here, we analyzed whether other WTA glycosylations could also promote *Lm* resistance against AMPs. To evaluate the role of WTA-GlcNAc in *Lm* Sv1/2a resistance to AMPs, we assessed the in vitro survival of *Lm* Δ*lmo1079*, as compared with the WT and Δ*rmlT* strains, in the presence of biologically active human LL-37 and its mouse homologue cathelicidin-related antimicrobial peptide (CRAMP; Figure 2 and Figure S1B). After two hours of incubation with AMPs, the surviving bacteria were quantified by colony-forming unit CFU counting (Figure 2A,C). Compared with the WT strain, the Δ*lmo1079* mutant displayed a slight increased susceptibility to CRAMP as the Δ*rmlT* strain (Figure 2A, upper left panel). Interestingly, the double mutant Δ*lmo1079*Δ*rmlT,* lacking both Rha and GlcNAc, displayed a significant increased susceptibility to both CRAMP and LL-37 (Figure 2A, right panels), indicating that WTA-Rha and WTA-GlcNAc cooperate to confer *Lm* resistance to AMPs. To further corroborate these data, the growth of *Lm* WT, Δ*lmo1079* and Δ*lmo1079*Δ*rmlT* strains, in the presence of CRAMP or LL-37 (10 μg/mL) was monitored over time through optical density measurement (Figure 2B). According to the survival data, we observed a slight but significant growth defect for the Δ*lmo1079* strain in the presence of CRAMP. In turn, the double Δ*lmo1079*Δ*rmlT* mutant showed a significant growth defect in the presence of CRAMP or LL-37, confirming that both Rha and GlcNAc of the *Lm* WTAs are important to confer resistance to AMPs.

The role of Gal and Glu in the resistance of the *Lm* Sv4b strain against AMPs was also assessed. Similar to the Sv1/2a strain, incubation with CRAMP only induced a slight decrease in the survival levels of single mutants (Δ*gttA* and Δ*gltB*). LL-37 had no effect on the survival of Δ*gttA* and Δ*gltB* strains, as compared with WT (Figure 2C). However, the double mutant Δ*gttA*Δ*gltB,* lacking both WTA-Glu and -Gal, displayed a dramatic increase in susceptibility to both CRAMP and LL-37 (Figure 2C).

In agreement, besides slight differences observed in the bacterial growth of single mutants in the presence of 10 μg/mL of AMPs, the double mutant exhibited clear growth defects in the presence of both AMPs, as compared with the WT strain (Figure 2D).

Altogether, these results demonstrate the role of different WTA glycosylations in the susceptibility to AMPs of diverse *Lm* serovars, and highlight the cooperative role of distinct glycosylations.

### 2.3. WTA-glycosylation Promotes *Lm* Decreased Susceptibility to Some Antibiotics

Then, we analyzed the potential role of WTA glycosylations in *Lm* susceptibility to antibiotics. We tested β-lactams (ampicillin and penicillin) and aminoglycoside (gentamicin), which are antibiotics commonly used in clinics for the treatment of human listeriosis [25].

**Figure 3:**
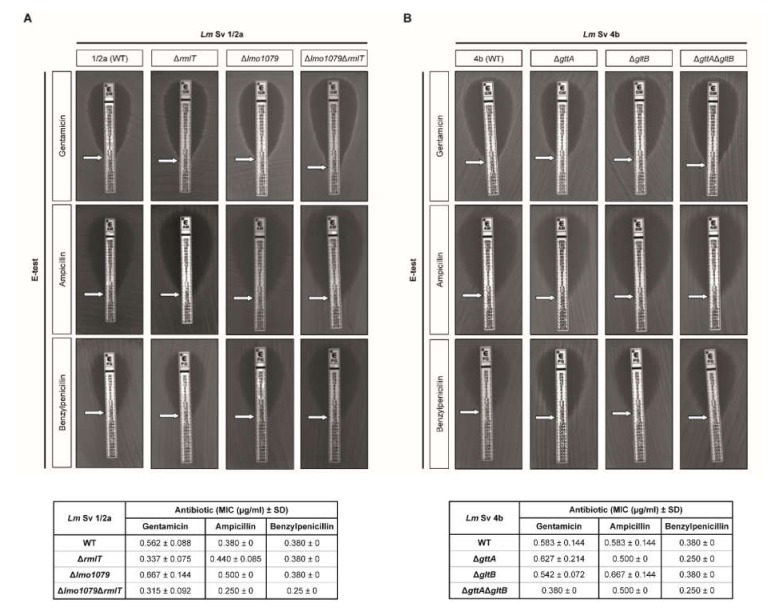
WTA-glycosylation promotes *Lm*-decreased susceptibility to antibiotics. E-test for gentamicin, ampicillin, and benzylpenicillin of *Lm* strains from (**A**) Sv1/2a and (**B**) Sv4b. The respective minimum inhibitory concentrations (MICs) are shown in the tables below the E-test images. Data represent mean ± SD of at least three independent experiments.

Minimum inhibitory concentrations (MICs) were determined by the Epsilometer test (E-test) method. *Lm* Sv1/2a and Sv4b showed a similar susceptibility towards penicillin and gentamicin (Figure 3A). However, *Lm* Sv4b appeared slightly less susceptible to ampicillin.

In the *Lm* Sv1/2a strain, the absence of WTA-Rha induced a slight increase in bacterial susceptibility to gentamicin. Remarkably, the double mutant lacking both Rha and GlcNac (Δ*lmo1079*Δ*rmlT*) showed an increased susceptibility to all of the antibiotics tested (Figure 3A). In the Sv4b strain, the simultaneous loss of Gal and Glu in WTAs (Δ*gttA*Δ*gltB* strain) also increased antibiotic sensitivity (Figure 3B).

Altogether, these results indicate that WTA glycosylations confer a specific decreased susceptibility to antibiotics, depending on the type of glycosylation and on the antibiotics, revealing the important role of WTA glycosylations in *Lm* susceptibility to antibiotics.

## 3. Discussion

While killing/inhibiting the growth of sensitive strains, conventional antibiotics enable resistant bacteria to grow in a competitor-free environment, creating strong selection for resistance. Rather than to kill or halt bacterial growth, one attractive strategy to disarm pathogens is by directly targeting virulence and/or resistance mechanisms using drugs that do not directly harm their targets, and thus do not impose resistance selection. Another promising approach is sensitizing bacteria from already existing drugs. The idea is to develop compounds that do not directly kill bacteria, but selectively disarm them, giving antibiotics the chance to act. Importantly, “disarm—do not kill” approaches also overcome two major antibiotics shortfalls, namely: drug resistance development and the killing of commensal bacteria.

Here, we demonstrated that WTA glycosylation appears to be a broad mechanism used by *Lm* not only to anchor virulence factors at its surface, but also to overcome the action host AMPs and antibiotics. Further studies should evaluate the role of WTA glycosylation in other *Lm* serovars.

To efficiently infect its host, *Lm* makes use of a large array of virulence effectors that act at different steps of the infection cycle [3]. *Lm* virulence factors include bacterial surface proteins that are associated with the cell wall. Their extra-cytoplasmic localization allows these proteins to interact directly with host cell targets, and to induce the effects necessary for the establishment of infection. The association of two of these virulence factors, Ami and InlB, at the bacterial surface was previously shown to be dependent on WTA-Rha [22]. Here, we demonstrate WTA glycosylation as a broad mechanism modulating proper protein association to the bacterial surface in diverse *Lm* Sv. In addition to WTA glycosylations, surface localization also depends on the protein itself. Ami and InlB belong to a protein family whose association to the bacterial surface is promoted by Glycine-Tryptophan (GW) modules. GW modules are sufficient for their WTA-Rha dependent surface localization; however, this is not the case for all *Lm* proteins containing GW modules [22]. Very recently, WTA-Gal was shown to be required for the stable surface association of another important *Lm* virulence factor, ActA, whose surface localization is unrelated to GW modules. The absence of WTA-Gal induces ActA secretion, and a concomitant loss of ActA-mediated intracellular motility and virulence [26]. This is in agreement with the decreased actin tail formation observed in a Sv4b WTA-Gal deficient mutant [23]. Altogether, these results point to a broad role of WTA glycosylations in the surface localization of *Lm* proteins involving diverse association systems.

AMPs are small peptides produced by living organisms [27], and constitute a major player of the innate immune response against microbial pathogens [28]. They interact with negatively charged prokaryotic surfaces, insert into the plasma membrane [27,29], and induce membrane disruption causing bacterial death [30]. Gram-positive pathogens have evolved counteracting strategies to avoid AMP-mediated killing, which include the modification of their surface charge by the D-alanylation of teichoic acids [31]. WTA glycosylations have also been shown as strategies used by *Lm* to resist to the action of AMPs [17,26]. Here, we show the cooperative action of several glycosylations in resistance to AMPs. While the absence of individual glycosylations (Rha and GlcNAc in Sv1/2a and Gal and Glu in Sv4b) only had a slight effect on the *Lm* resistance to AMPs, in both Svs, the concomitant absence of both glycosylations induced a large decrease on the *Lm* capacity to cope with AMPs. This could be the result of the compensatory mechanisms deployed in the absence of a single glycosylation. These results also suggest that the effect is independent from the type of glycosylation, but rather correlates with a general outcome that can be related to the decreased compactness of the cell wall and the consequent increased AMP penetration in the absence of WTA glycosylation. Indeed, WTA glycosylations appear to act mainly on the packing density and spatial constraints of the bacterial cell wall, thus modifying the kinetic of AMPs progression. This could induce some morphological alterations of the bacterial cell wall. However, our previous transmission electron microscopy analysis did not reveal any differences between the WT strain and a WTA-rhamnosylation deficient mutant [17].

We previously demonstrated that, because of their important role in the surface association of virulence factors and in resistance to AMPs, the absence of some WTA glycosylations have a strong impact on the *Lm* infectious capacity [17,23]. We show here that other WTA glycosylations share the same properties, however their role in virulence in vivo needs to be demonstrated.

In *Bacillus subtilis* and *S. aureus*, the loss of WTAs [32] or WTA β-GlcNAc modifications [24] renders bacteria sensitive to β-lactam antibiotics. Similarly, we found that the absence of WTA glycosylation induces an increased susceptibility of *Lm* to antibiotics, including ampicillin, penicillin, and gentamicin. Interestingly, as observed for AMPs, the simultaneous absence of several glycosylations induced an additional decrease in the capacity of *Lm* to growth in the presence of antibiotics. We can speculate that, as suggested for AMPs, this increased susceptibility to antibiotics could be due to an increased permeability of the PGN. In *S. aureus*, WTA-β-GlcNAc was proposed to sensitize bacteria to β-lactam antibiotics by scaffolding the proteins associated to the PGN [24], and *S. aureus* WTAs were shown to specifically interact with a penicillin-binding protein involved in the resistance to β-lactams [33]. In addition, WTA glycosyl substituents are also receptors for *Lm* phage proteins [34,35]. We thus propose that, as observed for virulence factors (Ami and InlB), WTA glycosylations could also be involved in the correct positioning of proteins involved in the decreased susceptibility to antibiotics. Most of the *Lm* strains are sensitive to antibiotics currently used in the treatment of human listeriosis, such as penicillin, ampicillin, and gentamicin. However, *Lm* strains isolated from food products appear to be resistant to some of these antibiotics [36]. Thus, it is important to analyze the role of WTA glycosylation in such resistant *Lm* strains.

We are currently in a race to develop new antimicrobials. While traditional antibiotics kill/inhibit the growth of bacteria, creating strong selection for resistance mechanisms [37], anti-virulence drugs disarm the pathogen without impacting its viability or growth, generating weaker resistance selection [38]. Given that the loss of most of the WTA glycosylations does not impact bacterial growth, WTA substituents hold promise for antimicrobial drug targeting. In view of their importance for pathogenesis, resistance to AMPs, and decreased susceptibility to antibiotics, this is particularly the case for *Lm* WTA glycosylations.

## 4. Materials and Methods

### 4.1. Bacterial Strains and Growth Conditions

The bacterial strains used in this study are described in Table 1. *Listeria monocytogenes* (*Lm*) strains were cultured in a brain heart infusion (BHI) medium (Difco), and *Escherichia coli* were grown in lysogeny broth (LB) media, both at 37 °C, with agitation. When necessary, the antibiotics ampicillin (amp) 100 μg/mL and erythromycin (ery) 5 μg/mL were added to select *E. coli* or *Lm*, respectively.

### 4.2. Construction of Deletion Mutant Strains

For this study, *Lm* mutant strains were constructed in a *Lm* Sv1/2a EGDe background through a double homologous recombination process mediated by the plasmid pMAD [39], as described in the literature [40]. DNA fragments corresponding to the upstream (UP) and downstream (DW) flanking regions of the target genes were amplified by a PCR from *Lm* EGDe chromosomal DNA, using specific primers 1–2 and 3–4 or 5–6, listed in Table 2.

**Table 1:** Bacterial strains and plasmids used in this study.

Bacterial Strains and Plasmid	Lab Code	Relevant Characteristics	Source
*L. monocytogenes*			
**EGD-e**	DC 4	Wild-type; Sv 1/2a	[41]
**EGD-e ∆*rmlT***	DC492	EGD-e *rmlT* (*lmo1080*) deletion mutant	[17]
**EGD-e ∆*lmo1079***	DC 858	EGD-e *lmo1079* deletion mutant	This study
**EGD-e ∆*lmo1079*∆*rmlT***	DC 899	EGD-e *lmo1079-lmo1080* deletion mutant	This study
**WSLC 1042 WT**	DC 825	Wild-type; Sv 4b	ATCC®23074
**WSLC 1042 ∆*gttA***	DC 826	WSLC 1042 *gttA* deletion mutant	[23]
**WSLC 1042 ∆*gltB***	DC 827	WSLC 1042 *gltB* deletion mutant	[23]
**WSLC 1042 ∆*gttA*∆*gltB***	DC 828	WSLC 1042 *gttAgltB* deletion mutant	[23]
***E. coli***			
**DH5α**		Competent cells	Life Technologies
**Plasmid**			
**pMAD**	DC 48	Amp^r^ and Ery^r^	[39]

**Table 2:** Primers used to amplify the flanking regions.

	Primers	Sequence (5’ → 3’) *	Restriction Enzymes
**1**	*lmo1079* UP Fw	AGTC**GGATCC**GGAGCATCTTCTACATTAGGC	BamHI
**2**	*lmo1079* UP Rv	AGTC**GTCGA**CCCATTAACTTTCTCCCTCC	SalI
**3**	*lmo1079* DW Fw	AGTC**GTCGAC**TAAATGAGGGAAAACGTTAGG	SalI
**4**	*lmo1079* DW Rv	AGTC**CCATGG**CACCGTGAATGAACGCC	NcoI
**5**	*lmo1080* DW Fw	CGG**GTCGAC**TAAGAATGGAGAGAAAAGAATGAAAGG	SalI
**6**	*lmo1080* DW Rv	CGG**CCATGG**GGAATGCTTTTTCATTATAGC	NcoI
**Internal Primers**			
**7**	*lmo1079* Fw	GCAAATTGGAATGGGAGGCG	
**8**	*lmo1079* Rv	GGATGCCTTGTTGCCGAAAC	
**9**	*lmo1080* Fw	TATTGCCACACGCTTTACCG	
**10**	*lmo1080* Rv	CTTCCACGATTGAACGAACG	
**11**	*lmo1492* Fw	GACGGATCCCGCAACTTCGCAAAATGGG	
**12**	*lmo1492 Rv*	AGCGTCGACGTCGCCATACCATCTGTTTG	
**pMAD Primers**			
**13**	pMAD Fw	TGATGGTCGTCATCTACCTGCC	
**14**	pMAD Rv	CCTACGTAGGATCGATCCGACC	

* Restriction sites added to the 5’ end of the primer sequence are underlined.

The purified fragments were digested with the corresponding restriction enzymes (BamHI-SalI and SalI-NcoI) and were colligated in the multiple cloning site of the digested plasmid pMAD. The plasmid constructs were electroporated into electrocompetent *Lm* EGD-e cells and the transformed bacteria were selected at 30 °C in the presence of ery 5 μg/mL. Positive clones were re-isolated on a BHI-ery 5 μg/mL and incubated at 43 °C. Ery-resistant colonies were inoculated in the BHI broth at 30 °C, diluted several times, plated in BHI, and incubated overnight at 37 °C. Individual colonies were grown on BHI and BHI ery agar plates, and erythromycin-sensitive colonies were screened for the absence of the target genes using PCR with internal primers (Table 2). Both plasmid constructs and *Lm* gene deletions were confirmed by DNA sequencing.

### 4.3. Growth Analysis in Vitro

Overnight cultures of the different strains were diluted to 1:100 in fresh BHI broth, and were cultured at 37 °C with shaking. Bacterial growth was followed by measuring the optical density at 600 nm of the bacterial cultures, every 45 minutes.

### 4.4. Extracts of Lm Proteins

The extraction of non-covalently surface-associated and secreted Lm proteins was performed as describe before [22]. Exponential phase bacteria (20 mL, OD600 ≈ 0.8) were pelleted by centrifugation (3800 g, 15 min, 4 °C), and both the pellet and the supernatant fractions were collected. Pellets were washed with ice-cold PBS (3800 g, 10 min, 4 °C), resuspended in 200 μL of PBS + 2% sodium dodecyl sulphate (SDS), incubated (30 min at 37 °C), and centrifuged (21,000 g, 1 min). Supernatants containing the solubilized non-covalently cell surface-bound proteins were analyzed by Western blot. The culture supernatant fractions were filtered (0.22 μm) and the secreted proteins were precipitated with 0.2 mg/mL of sodium deoxycholate (DOC; 30 min, 4 °C), followed by 6% trichloroacetic acid (TCA; 2 h, 4 °C). The precipitated proteins were recovered by centrifugation (13,400 g, 15 min, 4 °C) and were washed twice with cold acetone (100%). The pellet was air-dried and resuspended in 200 μL of 20 mM Tris-HCl, pH 7.4, for the Western blot analysis.

### 4.5. SDS-PAGE and Western Blot Analysis of Protein Extracts

*Lm* protein extracts were resolved by SDS-PAGE in an 8% polyacrylamide gel. The proteins were transferred (Trans-Blot Turbo Transfer System; Bio-Rad Laboratories) onto a nitrocellulose membrane, according the manufacturer’s guidelines. Nitrocellulose membranes were stained with Ponceau S for loading control and were blocked in Buffer A (20 mM Tris-HCl, 0.9% NaCl, pH = 7.4) with 0.1% tween and 5% skimmed milk. The membranes were incubated overnight at 4 °C with a primary antibody diluted in Buffer A with 0.1% tween and 2.5% skimmed milk—mouse monoclonal anti-InlB (A13.1 [42]; (1:2000)), rabbit polyclonal anti-Ami antiserum (R5, from Pascale Cossart, Institut Pasteur; 1:5000), and rabbit polyclonal anti-*Lm* Glyceraldehyde-3-phosphate dehydrogenase (GAPDH) (Abgent; 1:100). The membranes were probed with anti-mouse or anti-rabbit horseradish peroxidase (HRP)-conjugated secondary antibodies (1:2000; P.A.R.I.S Biotech) in Buffer A with 0.1% tween and 2.5% skimmed milk. The signals were detected by chemiluminescence using a PierceTM Western Blotting Substract kit (Thermo Fisher Scientific) under ChemiDoc XRS + equipment (Bio-Rad Laboratories).

### 4.6. Antimicrobial Peptides Susceptibility

WTA glycosylation deficient strains and isogenic wild type strains were tested for their susceptibility to LL-37 or CRAMP (AnaSpec), as described in the literature [17]. Briefly, bacteria in the exponential phase of growth (OD600 ≈ 0.8) were diluted to 10^4^ CFU/mL in a sterile phosphate buffer (PB) medium (10 mM phosphate buffer, pH 7.4; 1% BHI). Bacterial suspensions were incubated with CRAMP or LL-37 in a 96-well microplate (2 h at 37 °C), without shaking, serially diluted in sterile phosphate buffer solution (PBS), and plated in BHI agar for the quantification of viable bacteria. As the control, bacterial suspensions without AMPs were used. For growth analysis in the presence or absence of AMPs, *Lm* strains in the exponential phase of growth (OD600 ≈ 0.8) were diluted (10^7^ CFU/mL) in a sterile PB medium in a 96-well microplate, and incubated with or without CRAMP or LL-37 for 4 h at 37 °C with shaking. The optical densities at 600 nm were measured every 15 minutes using the Synergy2 equipment (BioTek). Solutions containing only AMPs were used for background subtraction.

### 4.7. Antibiotic Susceptibility

The susceptibility to antibiotics of *Lm* strains was assessed by measuring the MICs through the E-test method (Table 3). Bacteria in the stationary phase were inoculated at a high density on Mueller Hinton (MH) agar (Sigma-Aldrich) plates with a sterile cotton swab. ETEST® strips (BioMérieux) of each antibiotic (Table 3) were applied on the inoculated plates before overnight incubation at 37 °C. The day after, the susceptibility halos were analyzed as recommended.

### 4.8. Statistical Analysis

Statistical analyses were carried out with Prism software (GraphPad) using one-way ANOVA with Dunnett’s post hoc analyses to compare different means in relation to a control sample, and Tukey’s post hoc analyses for pairwise comparisons of more than two different means. A two-tailed unpaired Student’s t-test was used for a comparison of the means between two samples. For statistically significant differences: * *p* ≤ 0.05; ** *p* ≤ 0.01; *** *p* ≤ 0.001; **** *p* ≤ 0.0001.

## Figures and Tables

**Table 3:** Range of antibiotic concentrations tested against *Lm*.

Antibiotic	Strip Concentration (μg/mL)
**Gentamicin** **Ampicillin** **Benzylpenicillin**	0.016-256 0.016-256 0.002-32

## Supplementary Materials

The following are available online at https://www.mdpi.com/2076-0817/9/4/290/s1, Figure S1: Restoration of WT phenotypes for the *ΔrmlT* mutant after re-introducing the *rmlT* gene.
