# Supplementary material for: Listeria monocytogenes Wall Teichoic Acid Glycosylation Promotes Surface Anchoring of Virulence Factors, Resistance to Antimicrobial Peptides, and Decreased Susceptibility to Antibiotics
Source: Pathogens. 2020 Apr 16;9(4):290. doi: 10.3390/pathogens9040290 (PMC7238011; doi:10.3390/pathogens9040290)
Supplement: Supplementary file 1 [file pathogens-09-00290-s001.pdf]

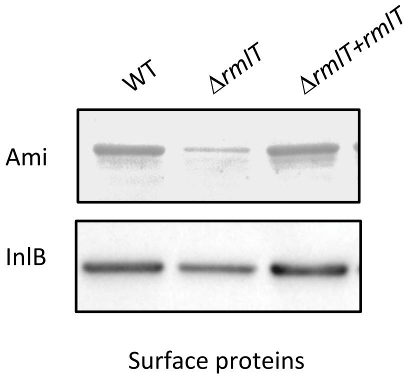

WTA-glycosylation promotes surface association of *Lm* virulence factors. Western blot on extracts of non-covalently cell surface associated *Lm* proteins obtained from WT, Δ*rmlT* and Δ*rmlT+rmlT* strains. Images are representative of at least three independent experiments.

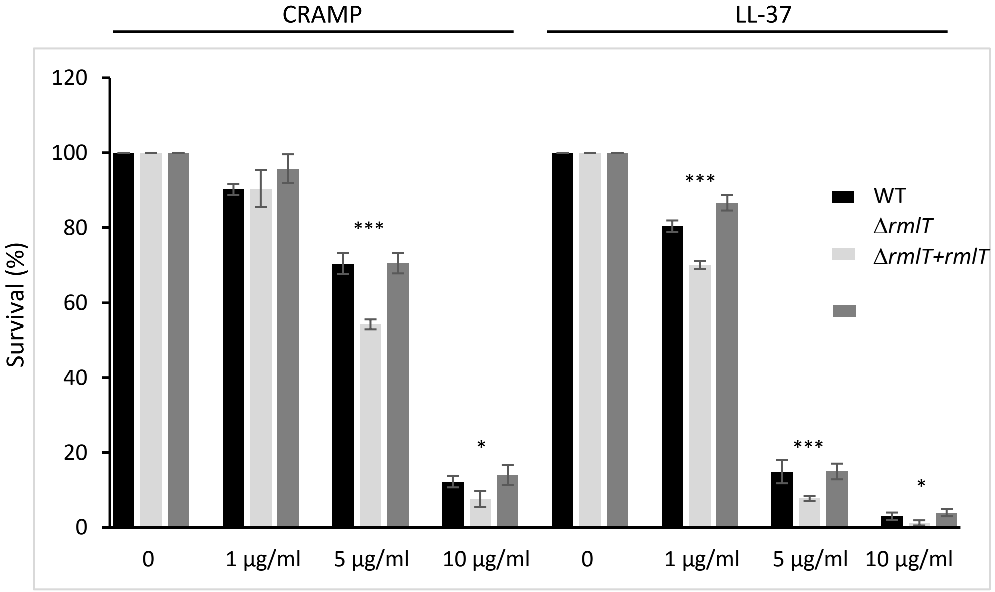

WTA-glycosylation promotes *Lm* resistance against AMPs. Quantification of viable *Lm* after incubation of exponential-phase WT, Δ*rmlT* and Δ*rmlT+rmlT* strains with the antimicrobial peptides CRAMP and LL-37. Values from AMP-treated samples were normalized to untreated controls (set at 100). Data represent mean±SD of at least three independent experiments. (*, $p < 0.05$; ***, $p < 0.001$).

Figure S1. Restoration of WT phenotypes for the Δ*rmlT* mutant after re-introducing the *rmlT* gene.
